# Whole blood and blood components from vertebrates differentially affect egg formation in three species of anautogenous mosquitoes

**DOI:** 10.1186/s13071-021-04594-9

**Published:** 2021-02-24

**Authors:** Ruby E. Harrison, Mark R. Brown, Michael R. Strand

**Affiliations:** Department of Entomology, The University of Georgia, 120 Cedar Street, 420 Biological Sciences, Athens, GA 30602 USA

**Keywords:** Mosquito, Oogenesis, Reproduction, Endocrinology, Diet

## Abstract

**Background:**

Most female mosquitoes are anautogenous and must blood feed on a vertebrate host to produce eggs. Prior studies show that the number of eggs females lay per clutch correlates with the volume of blood ingested and that protein is the most important macronutrient for egg formation. In contrast, how whole blood, blood fractions and specific blood proteins from different vertebrates affect egg formation is less clear. Since egg formation is best understood in *Aedes aegypti,* we examined how blood and blood components from different vertebrates affect this species and two others: the malaria vector *Anopheles gambiae* and arbovirus vector *Culex quinquefasciatus*.

**Methods:**

Adult female mosquitoes were fed blood, blood fractions and purified major blood proteins from different vertebrate hosts. Markers of reproductive response including ovary ecdysteroidogenesis, yolk deposition into oocytes and number of mature eggs produced were measured.

**Results:**

*Ae. aegypti, An. gambiae* and *C. quinquefasciatus* responded differently to meals of whole blood, plasma or blood cells from human, rat, chicken and turkey hosts. We observed more similarities between the anthropophiles *Ae. aegypti* and *An. gambiae* than the ornithophile *C. quinquefasciatus.* Focusing on *Ae. aegypti,* the major plasma-derived proteins (serum albumin, fibrinogen and globulins) differentially stimulated egg formation as a function of vertebrate host source. The major blood cell protein, hemoglobin, stimulated yolk deposition when from pigs but not humans, cows or sheep. Serum albumins from different vertebrates also variably affected egg formation. Bovine serum albumin (BSA) stimulated ovary ecdysteroidogenesis, but more weakly induced digestive enzyme activities than whole blood. In contrast, BSA-derived peptides and free amino acids had no stimulatory effects on ecdysteroidogenesis or yolk deposition into oocytes.

**Conclusions:**

Whole blood, blood fractions and specific blood proteins supported egg formation in three species of anautogenous mosquitoes but specific responses varied with the vertebrate source of the blood components tested.
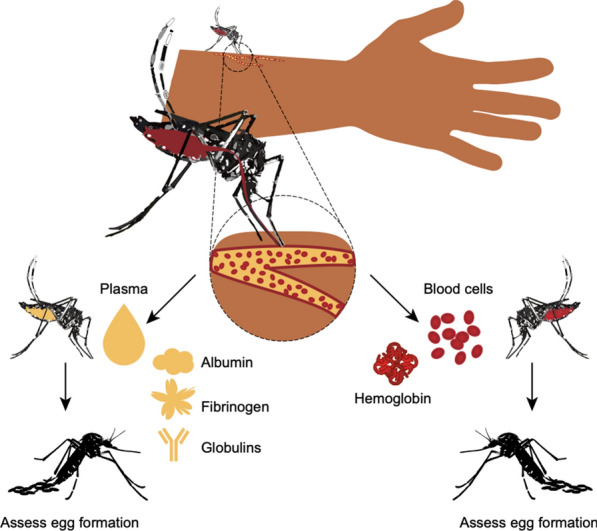

## Background

Mosquitoes vector a number of bloodborne pathogens, including the causative agents of malaria, dengue fever and yellow fever, that annually result in > 200 million human infections and 725,000 deaths worldwide [1]. Adult mosquitoes of both sexes can subsist entirely on nutritional resources such as nectar or fruit juices that primarily contain carbohydrates [2]. Plant sugars provide energy for maintenance, allow for metabolic reserve replenishment, extend life span and are used directly to fuel flight [3]. However, most species are also anautogenous, which means that females cannot produce eggs without consuming blood from a vertebrate host [4]. The ability to blood feed and produce eggs in consecutive cycles over a lifespan of 4–8 weeks further underlies how anautogenous mosquitoes acquire and transmit pathogens among vertebrate hosts [4].

Mature eggs develop in the ovaries from primary egg chambers comprised of an oocyte, nurse cells and enveloping follicle cells [5]. Primary egg chambers remain developmentally arrested until consumption of a bloodmeal, which activates the vitellogenic phase of oogenesis [6]. The processes regulating the vitellogenic phase are currently best understood in *Aedes aegypti* where consumption of a bloodmeal stimulates the release of two types of peptide hormones, ovary ecdysteroidogenic hormone (OEH) and insulin-like peptides (ILPs), from the brain [6]. OEH and ILP family members such as ILP3 bind distinct but related receptor tyrosine kinases named the insulin (IR) and OEH (OEHR) receptors [7–9]. Ligand binding activates the IR and OEHR on ovary membranes, which activates the division of follicle cells and endoreplication of nurse cells [5]. OEH strongly stimulates follicle cells to produce ecdysteroid hormones, chiefly ecdysone (ECD) [8–11], while ILP3 is required for expression of trypsin-like enzymes in the midgut that digest the bloodmeal [12–15]. ECD released from follicle cells is converted to 20-hydroxyecdysone (20E) in fat body adipocytes, which together with nutrient signaling via the target of rapamycin (TOR) pathway stimulates the synthesis of vitellogenin (Vg) and other yolk components [16]. Mature eggs are formed upon completion of yolk uptake and deposition of a chorion (egg shell) by follicle cells [17, 18]. Females then lay up to 120 eggs in a single clutch by 72 h post-bloodmeal [4].

Whole vertebrate blood is a colloidal suspension of predominantly red blood cells (erythrocytes) in plasma. Whole blood also consists primarily of protein by dry weight, with only small amounts of lipid, carbohydrate and trace elements [19]. However, hematological parameters also vary among vertebrates with significant differences observed among species in red blood cell diameter, hematocrit (packed cell volume), hemoglobin level, plasma protein content and salt, mineral and micronutrient concentrations [20–22]. Intraspecific variation is also observed among individual hosts because factors such as age, sex and health influence hematological properties [23]. These sources of variation in blood composition have potentially important consequences for mosquitoes since the nutritional quality of blood has been previously implicated in reproductive fitness [24–26].

Several studies indicate that anautogenous mosquitoes produce eggs after consuming blood from different vertebrates [26–42] with clutch size correlating with blood volume ingested [32, 43–47] and protein being the most important macronutrient [48–52]. Reviews of the early literature suggest anautogenous mosquitoes produce more eggs after blood feeding on amphibians, reptiles or birds than on mammals [36, 53]. Amino acid balance and the nucleated erythrocytes of amphibians, reptiles and birds have both been suggested to be more suitable for egg formation than the amino acid composition and anucleate erythrocytes of mammalian blood [31, 36, 54, 55]. The packed cell volume of blood has been shown to influence reproductive fitness, survival and vectorial capacity in mosquito species that undergo prediuresis, i.e. that concentrate red blood cells in the midgut and excrete plasma continuously while blood-feeding [56–60]. Some studies indicate the cellular fraction of blood from different host species contributes to or is indispensable for egg formation in several anautogenous species including *Ae. aegypti* [36, 49, 55, 58, 61–64]. In contrast, a recent study of bovine blood fractions concluded that only plasma supported egg formation by *Ae. aegypti* [65], and lyophilized plasma forms a suitable artificial diet for *Aedes* and *Anopheles* mosquitoes [66]. The most abundant protein in bovine plasma, serum albumin, has also been shown to stimulate high levels of egg formation in *Ae. aegypti* and select other species [65, 67–74].

In assessing the preceding literature, we noted that most studies focus on single mosquito species and often use differing assay approaches, which could contribute to varying conclusions about how blood and blood components from different vertebrates affect egg formation. We also noted that most studies do not examine how blood components affect key physiological processes that regulate vitellogenesis. Since egg formation is best understood in *Ae. aegypti,* we revisited how blood and blood components from different vertebrates affect this species and two others: the malaria vector *Anopheles gambiae* and arbovirus vector *Culex quinquefasciatus*. In the first part of our study, we assessed feeding rates, yolk deposition, the number of eggs laid and hatch rates in these three mosquito species when fed whole blood or blood fractions from different vertebrates. We then focused the second part of our study on *Ae. aegypti,* where we investigated how blood-derived and other proteins affect yolk deposition, digestive enzyme expression, and ovary ecdysteroidogenesis. Our results indicate that plasma, blood cells and particular proteins stimulate mosquito egg formation, but outcomes differ with the vertebrate source of the components tested.

## Methods

### Mosquito rearing

The University of Georgia (UGAL) strain of *Aedes aegypti* was established from wild-caught females in Athens, GA, in the early 1970s. Morphological characters identify this strain as the subspecies *Aedes aegypti aegypti* [75], but for brevity we henceforth refer to the UGAL strain as *Ae. aegypti.* The G3 strain of *Anopheles gambiae* was originally obtained from the Centers for Disease Control and Prevention (CDC) in Atlanta and has been maintained in our insectary since 2004. The CDC MR4/BEI strain of *Culex quinquefasciatus* has been maintained in our insectary since 2011. All mosquitoes were reared under a 12 h light:12 h dark photoperiod at 26 °C and 70% relative humidity. Larvae of each species were reared in pans at a density of ~150 larvae per liter of deionized water and fed daily until pupation at 6 days post-hatch. *Ae. aegypti* and *C. quinquefasciatus* were maintained on a larval diet consisting of ground rat chow pellets (LabDiet 5001), lactalbumin (Sigma) and torula yeast extract (Bio-Serve) mixed in a ratio of 1:1:1 by volume, referred to as rat chow mix. *An. gambiae* larvae were maintained on a diet of pulverized TetraMin tropical flakes (Tetra). Adult mosquitoes were provided water and 10% sucrose in water *ad libitum*. For colony maintenance, adults from each generation were blood-fed 3–5 days post-eclosion to obtain eggs. *Ae. aegypti* and *C. quinquefasciatus* were blood-fed from an anesthetized male laboratory rat (Sprague Dawley), while *An. gambiae* was fed defibrinated rabbit blood (Hemostat Laboratories) using a membrane feeder.

### Membrane feeding

**Fig. 1:**
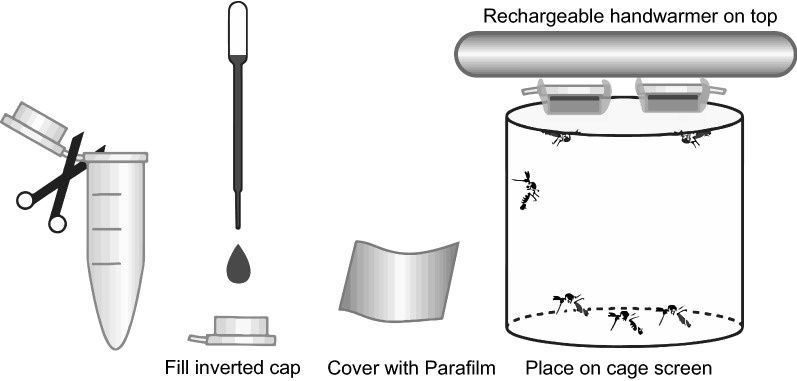
Schematic illustrating the membrane feeder system used during the study. See "Methods" for details

Membrane feeder assays were conducted using a simple method that has not previously been reported in the literature. In brief, meals were pipetted into the cut-off caps of 1.7-ml microfuge tubes (Olympus Plastics 22-282), which hold a 200-µl volume. Parafilm® M (Millipore Sigma P7543) was stretched over these caps to produce small capacity membrane feeders. Four to eight caps, depending on the number of mosquitoes fed (ranging 15–50), were then placed on the mesh tops of small mosquito cages (10 × 8.5 cm), membrane side down, and topped with a USB-chargeable hand warmer from either Thermotrek© or FourHeart® (Fig. 1). When operated at the lowest heating level, these electric handwarmers maintain stable temperatures of 40 and 42 °C, respectively.

All whole blood, blood fraction and protein meals contained ATP at a final concentration of 1 mM, which is a potent phagostimulant at this concentration [71, 76]. Whole bloods used in membrane feeder assays were purchased from Hemostat Laboratories (Dixon, CA) and contained the anticoagulant sodium citrate unless otherwise specified. Due to the rapid expiration of bottled blood, whole blood and blood fractions were always offered to mosquitoes within 4 days of blood shipment arrival. Whole bloods were centrifuged at 2000 × *g* for 5 min to produce plasma and cell fractions that primarily consisted of erythrocytes. Undiluted plasma was put into membrane feeders while cell fractions were triple-washed in phosphate-buffered saline (PBS) [65] and then resuspended at a hematocrit of 50% in fresh PBS, which approximated the mean packed cell volume determined for many mammalian and avian species [21]. All proteins, peptides and amino acids used in assays were commercially purchased (Sigma Aldrich, St. Louis, MO; Difco, Detroit, MI) and fed at a concentration of 200 mg/ml in PBS plus ATP. An L-amino acid solution containing the 20 amino acids present in most eukaryotic proteins was prepared in PBS at molarities that matched the amino acid content of BSA at 200 mg/ml with the exception of tyrosine, which was reduced to 50% of BSA to fully solubilize (Additional file 1: Table S1).

### Bioassays

To estimate the amount of blood mosquitoes consumed, 4-day post-emergence females were chilled and weighed in cohorts of ten using an analytical balance (Ohaus DV215CD). The average weight per individual NBF female was calculated from this value. Each cohort of ten was returned to a cage with access to water for 8 h and then presented membrane feeders containing different solutions for 30 min. Immediately following blood-feeding, females were then chilled and the replete individuals tallied and weighed, which yielded an average bloodmeal weight per individual (mg). Bloodmeal weights were always taken within 15 min of blood-feeding since *Ae. aegypti* females undergo diuresis and excretion of excess blood-derived water during the first hour post-bloodmeal [4]. Each treatment was replicated ten times.

To assess clutch size, replete females were isolated within 1 h post-meal and individually placed in small cages (34 × 30 mm^2^) containing a paper towel-lined glass bowl half-filled with deionized water for oviposition. Glass bowls were checked for eggs daily and retrieved once oviposition had occurred or after 7 days post-meal. Clutch size was recorded as the number of eggs laid, while viability was calculated as the number of viable larvae produced per female. *Ae. aegypti* females laid eggs on the paper towel lining, which was dried and kept in a humid container for 10 days prior to hatching. *C. quinquefasciatus* and *An. gambiae* oviposit directly on the surface of the water; eggs from these species hatched within 72 h of oviposition at which time larvae were counted.

Oocyte maturation as measured by yolk deposition was measured using previously established methods [7, 77]. Briefly, ovaries were dissected from replete females 48 h after feeding, and the length of visible yolk per oocyte along the anterior-posterior access was measured using an ocular micrometer mounted on a stereomicroscope. For each female, measurements were taken for three oocytes per ovary pair, and averaged. Yolk deposition per female at 48 h post-meal was measured and averaged for 20–50 individual females per treatment.

### Ecdysteroid assays

Total ecdysteroids secreted by ovaries were quantified using an enzyme-linked immunosorbent assay (EIA) [10]. Ovaries were dissected from females 20 h after feeding, which corresponds with when ecdysteroid production peaks in ovaries after consumption of rat blood [8]. The ovaries from two females were pooled and incubated together in 60 µl Beyenbach’s saline [10] on a shaker for 6 h at 27 °C and ~90% RH. Following this incubation period, medium supernatant containing secreted ecdysteroids was collected and frozen at – 20 °C. Ecdysteroid titer was then determined by EIA using the EAB27 primary antibody and 20-hydroxyecdysone (Sigma) as a standard [78].

### Midgut trypsin-like serine protease activity and relative quantitative polymerase chain reaction (rqRT-PCR) assays

Trypsin-like serine protease activity was measured in midguts dissected from females 24 h after feeding using an established *N*_α_-benzoyl-L-arginine 4-nitroanilide hydrochloride (BApNA) assay [15]. In brief, each midgut was placed in Tris HCl 0.02 M buffer containing CaCl_2_ 0.02 M (200 µl), sonicated briefly, and then centrifuged at 12,000 × *g* for 5 min. Ten µl of supernatant (0.05 midgut equivalent) was then added to 90 µl Tris-CaCl_2_ buffer and 200 µl of BApNA (4 mM) (Sigma). After incubating on a rocker for 15 min at room temperature, absorbance was read at 405 nm (BioTek plate reader) and quantified based on trypsin standards (bovine pancreas, Sigma).

For rqRT-PCR assays, midguts were collected 24 h after females fed to repletion on membrane feeders and extracted using Trizol reagent (Ambion). Samples consisted of pooled midguts of two females, with four biological replicates per treatment. cDNA templates were generated using the iScript cDNA synthesis kit (Bio-Rad) while gene-specific primers were designed and purchased (IDT) for the following targets: *Aedes aegypti 5G1* (*Aa5G1*, X64363.1; forward 5’-CTGTGGAGGATCGCTACTTTC-3’; reverse 5’- GATGGCGGTTGACCTTCTTA-3’; *Aedes aegypti late trypsin* (*AaLT*, M77814.1; forward 5’-GGAAGTGATACCTTTACCGACCG-3’; reverse 5’-GATCACCAACGGGCTGTAGGC-3’), *Aedes aegypti serine protease VI* (*AaSPVI*, GQ398048; forward 5’-AGGAATGCCACAAGGCTTACTTGA-3’; reverse 5’-CCATAACCCCAGGATACCACT-3’); and *Aedes aegypti serine protease VII* (*AaSPVII*, GQ398049; forward 5’-CGAATGGTATGTGCCGGTTA-3’; reverse 5’-CAACTCCGACCAGGGTATTG-3’) [79]. *Aedes aegypti actin* (*AaACT,* KY000701) was used as reference gene using primers forward 5’-CGTTCGTGACATCAAGGAAA-3’ and reverse 5’-GAACGATGGCTGGAAGAGAG-3’ [79]. Reactions contained 3 µl cDNA, 2 µl forward/reverse primers 5 µM and 5 µl iQ SYBR Green Supermix (Bio-Rad 170-8882) and were run using a Rotor-Gene Q real-time PCR cycler (Qiagen) under the following conditions: denaturation at 95 °C for 10 s and annealing at 60 °C for 45 s, for a total of 30 cycles. Relative transcript abundance of each target gene relative to *AaACT* and fold change compared to non-fed controls was calculated using the ΔΔCT method [80].

### Data analysis

Individual adult females were the unit of replication in assays that assessed proportions of replete or ovipositing females, yolk deposition, time (days) taken to oviposit following ingestion of a meal and egg clutch size. Isolated midguts from individuals served as the unit of replication for trypsin activity assays. For feeding assays, the number of engorged females was summed across replicates and divided by the total offered the meal to generate an overall proportion of replete females.

All proportional data sets (e.g. proportion of females to blood-feed, proportion of females to oviposit, proportion of eggs to hatch) were assessed for significant differences among treatments using a contingency table analysis (chi-squared test). Continuous data such as yolk deposition, number of days taken to oviposit, egg clutch size and trypsin activity per midgut were analyzed for differences among treatment means using analysis of variance (ANOVA). The continuous-type data sets were initially assessed for normality using the Shapiro-Wilk W test on residuals and Bartlett’s test for homogeneity of variances. If both Shapiro-Wilk and Bartlett’s tests yielded non-significant *p*-values, treatment means were then compared using the parametric one-way ANOVA, followed by Tukey’s Honestly Significant Difference multiple comparisons post-hoc analysis. If the continuous data were found to be non-normal, a Kruskal-Wallis non-parametric alternative was used instead, followed by Dunn’s post-hoc multiple comparison test. Data on the weight of blood ingested, ecdysteroid production by ovaries and serine protease transcript profiles were generated from replicated samples of pooled females as previously described under Bioassays. Treatment means from these data sets were also analyzed by one-way ANOVA following the assessment of normality described for the other continuous data sets. All data analysis was performed using R, version 3.3.2. GUI 1.68 Mavericks build (7288). Tables were generated using Microsoft Excel v16.39 and figures generated using GraphPad Prism v8.4.2.

## Results

### UGAL strain *Aedes aegypti* similarly respond to blood feeding on a rat or membrane feeders containing rat blood

We first assessed whether females exhibited any differences in feeding, survival, oviposition or number of eggs laid when given an anesthetized rat versus membrane feeders filled with commercially purchased bottled blood. Because anticoagulants in blood have previously been shown to affect mosquito feeding success and survival [81], we compared mosquito responses to defibrinated rat blood or rat blood containing three different anticoagulants: Na citrate, Na heparin or EDTA. Proportionately fewer females fed to repletion after 30 min on a rat but average meal size (mg) was similar across treatments except for blood containing EDTA (Table 1). Survival over the course of these assays was unaffected by treatment since no replete females died. Days to oviposition were also similar among treatments except for females that consumed blood with EDTA, which oviposited approximately 1 day sooner than females that consumed blood from a rat (Table 1). No differences were detected in number of eggs laid (clutch sizes) among treatments although normalizing the data by bloodmeal size suggested females laid more eggs per mg of blood containing EDTA (Table 1). Overall, few differences were detected between females that fed on a rat and membrane feeders containing commercially purchased rat bloods. We thus used membrane feeders rather than living hosts to compare the effects of different bloods and blood components from different vertebrates on egg formation, using sodium citrate as the anticoagulant for all treatments.

**Table 1:** Feeding, oviposition time and eggs produced per *Ae. aegypti* female provided a live rat or bottled bloods in membrane feeders

	Live rat	Bottled rat blood with anticoagulant			
		Defibrinated	Na citrate	Na heparin	EDTA
Proportion of replete females^1^	46/100	67/100	66/100	61/100	59/100
Ingested blood (mg) per female	2.90±0.94^a^	2.45±0.32^a^	2.27±0.33^a^	2.15±0.64^a^	1.26±0.55^b^
Females examined for time to oviposition and eggs laid^2^	20	8	20	18	10
Time to oviposition (days)^3^	3.90 ± 0.85^a^	3.50 ± 1.41^a, b^	3.60 ± 0.50^a,b^	3.28 ± 0.9^a,b^	3.00 ± 0.00^b^
Total eggs per female	123.2 ± 48.0	140.6 ± 37.7	110.0 ± 44.7	115.3 ± 48.4	137.4 ± 25.1
Eggs per mg blood consumed^3^	46.7 ± 21.0^a^	57.9 ± 15.5^a, b^	49.2 ± 20.0^a^	57.6 ± 26.1^a,b^	69.0 ± 12.6^b^

^1^Females were fed in cohorts of 10 per cage for a total of 100 females per treatment. *χ*^2^ = 11.76, df= 4, *p*= 0.02

^2^A subsample of replete females was examined to determine time to oviposition, total eggs per female (clutch size) and eggs per mg of blood consumed

^3^For each indicated row, the mean ± SD for each treatment is indicated. Different small case letters after a given mean indicates treatments significantly differed from one another after ANOVA or Kruskal-Wallis and post hoc Tukey Kramer or Dunn’s tests, respectively

Different letters to the right of means in each row indicated the treatments significantly differed from one another (*p* ≤ 0.05).

### Whole blood, plasma and blood cells from four vertebrates differentially support egg formation

Multiple olfactory and visual cues have been implicated in attracting anautogenous female mosquitoes to vertebrates [82]. While most anautogenous mosquitoes are generalists that bite multiple vertebrates, *Ae. aegypti* shows evidence of evolving from a generalist feeder in its native Africa into two subspecies: *Ae. aegypti formosus*, which remains a generalist in Africa, and *Ae. aegypti aegypti,* such as the UGAL strain used in this study, which has been introduced worldwide and preferentially feeds on humans [25, 75]. Vertebrate blood is also known to differ among species with the protein content of plasma usually being higher in mammals than other taxa, while red blood cells vary from being anucleate in mammals to large and nucleate in birds, reptiles and amphibians [19]. However, it is largely unclear whether anautogenous species with host preferences derive reproductive benefits from the blood of preferred hosts. We therefore first asked whether females of each species differentially fed or laid eggs after feeding on commercially purchased bloods from two mammals (human, rat) and two birds (chicken, turkey) in no-choice membrane feeder assays that lacked most olfactory and visual cues associated with living hosts.

**Fig. 2:**
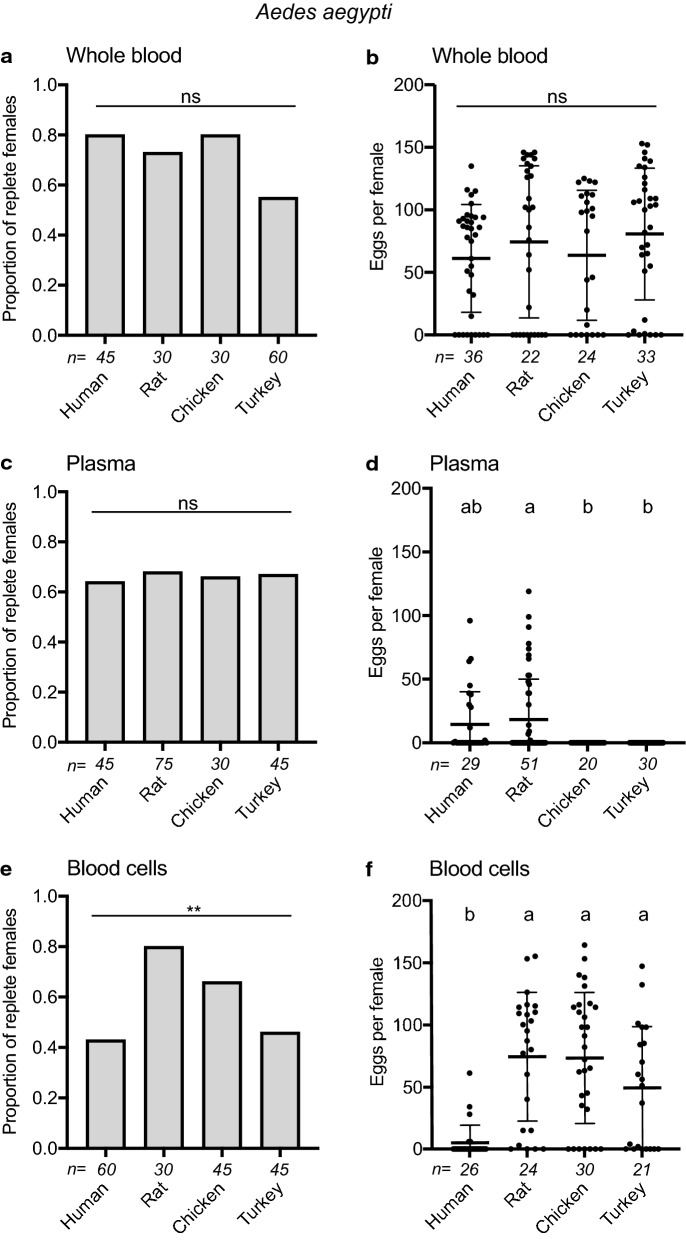
Feeding and egg laying by *Ae. aegypti* in response to blood and blood fractions from four vertebrates. Females were provided whole blood (**a**, **b**), plasma (**c**, **d**) or blood cells (**e**, **f**) containing ATP 1 mM in membrane feeders from human, rat, chicken or turkey. **a**, **c** and **e** Comparison of the proportion of females that fed to repletion. Numbers below the *x*-axis indicate the total number of individual females tested for a given treatment. Statistical significance after contingency table analysis is indicated by asterisks: *p < 0.05; **p < 0.001; ***p < 0.0001; *ns* not significant. **b**, **d**, and **f** Comparison of the number of eggs each female that fed to repletion laid. Numbers below the *x*-axis indicate the total number of individual replete females assessed for egg lay for each treatment. Horizontal bars indicate the mean ± SD. Within each graph different small case letters indicate treatments significantly differed from one another after a Kruskal-Wallis and post-hoc Dunn’s test (*p* ≤ 0.05)

We detected no differences among treatments in the proportion of UGAL *Ae. aegypti* females that fed to repletion or the number of eggs females laid (Fig. 2a, b). In contrast, large differences were detected in egg hatch rates with turkey blood producing the largest yields and chicken blood producing the smallest (Additional file 1: Table S2). We next separated each whole blood into its plasma and cell fraction. No differences were detected in the proportion of females that fed to repletion on each plasma (Fig. 2c), whereas large differences were detected in the number of eggs laid because of females producing none after feeding on bird plasmas (Fig. 2d). Egg hatch rates were also lower for females that consumed human versus rat plasma (Additional file 1: Table S2). Dissection of females that consumed avian plasmas showed that oocytes contained little yolk (ca. 40–100 µm), indicating that avian plasmas initiated vitellogenesis but did not sustain full maturation of oocytes. More females fed to repletion on rat than other blood cells (Fig. 2e), while females that consumed human blood cells laid no or very few eggs that also exhibited low hatch rates (Fig. 2f; Additional file 1: Table S2).

**Fig. 3:**
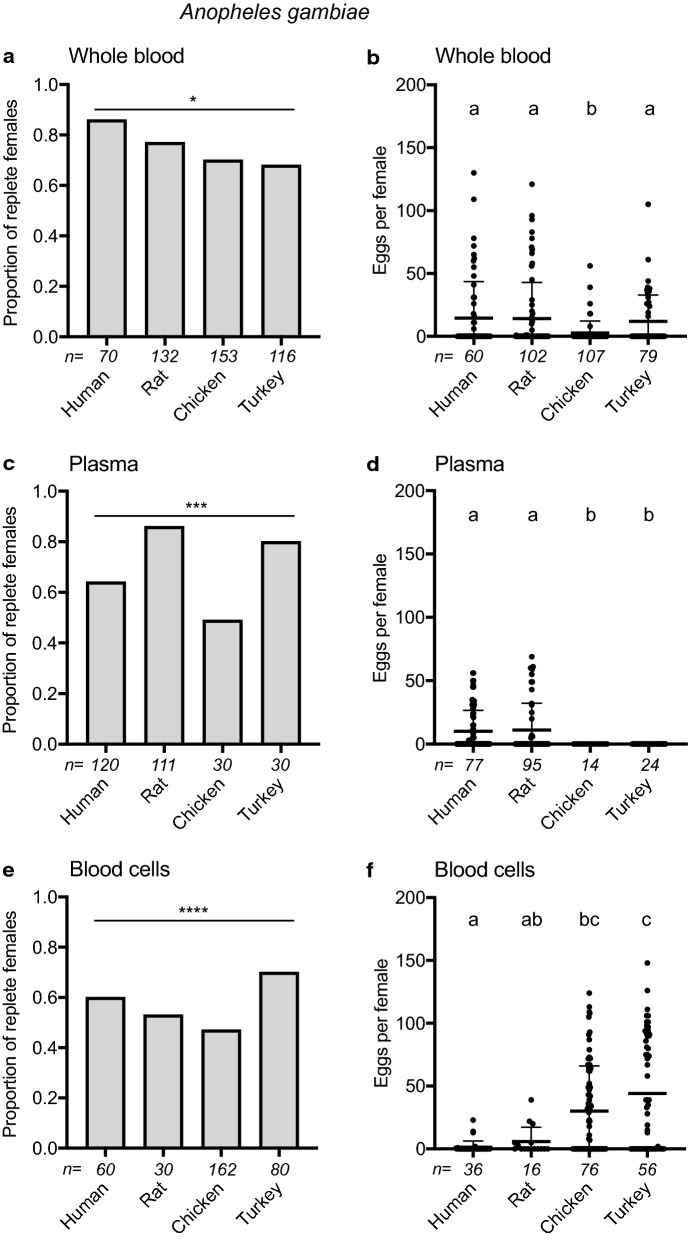
Feeding and egg laying by *An. gambiae* in response to vertebrate blood and blood fractions. Females were provided whole blood, plasma or blood cells from four vertebrates. Bar graphs on the left indicate the proportion of females that fed to repletion, with sample size (i.e., the number of individuals offered the meal) indicated below the *x*-axis in italics. Statistical significance after contingency table analysis is indicated (*p < 0.05; **p < 0.001; ***p < 0.0001; *ns* not significant). Dot plots on the right show the number of eggs laid by replete females, with sample sizes (i.e., number of replete females obtained from the feeds on the left panel) indicated below the x-axis and horizontal bars showing the treatment mean ± SD. Small case letters indicate treatments that significantly differed from one another after a Kruskal-Wallis and post-hoc Dunn’s test (*p* ≤ 0.05)

**Fig. 4:**
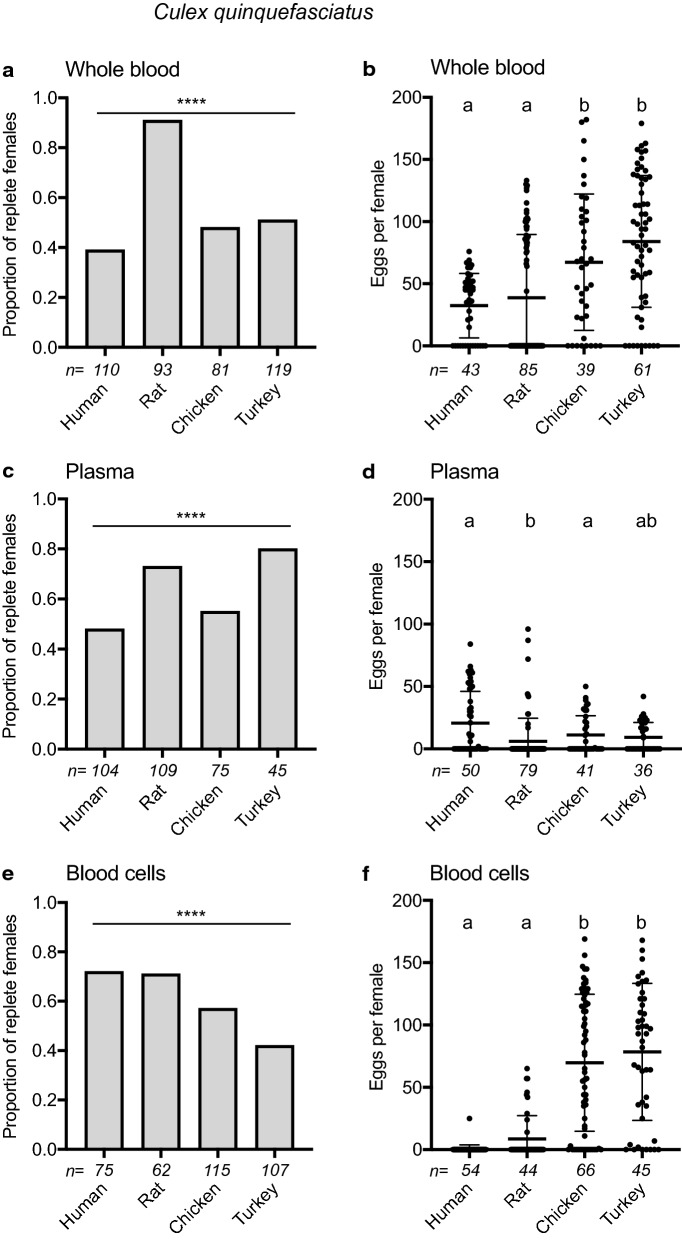
Feeding and egg laying by *C. quinquefasciatus* in response to vertebrate blood and blood fractions. Data are presented as in Figs. 2 and 3, with proportions of females fed to repletion indicated in **a**, **c** and **e** and egg clutch size per replete female shown in **b**, **d** and **f**. Italicized numbers below the x-axis indicate sample sizes, and statistical significances are indicated as previously described in the legends of Figs. 2 and 3

To assess whether the preceding outcomes were generalizable, the same treatments were tested with *An. gambiae,* which is strongly anthropophilic, and *C. quinquefasciatus*, which in the field preferentially feeds on birds but also feeds on several mammals including humans [83, 84]. The proportion of *An. gambiae* and *C. quinquefasciatus* that fed to repletion on whole blood, plasma or cell fractions varied among treatments but overall showed no tendencies in terms of *An. gambiae* more readily feeding on human and *C. quinquefasciatus* more readily feeding on avian-derived components (Figs. 3 and 4). *An. gambiae* laid the fewest eggs after consuming whole chicken blood; *C. quinquefasciatus* laid the most eggs on avian whole bloods, but hatch rates showed no tendencies in either species (Figs. 3 and 4, Additional file 1: Table S2). Broadly similar patterns to *Ae. aegypti* were observed when blood fractions were fed to *An. gambiae* with females laying no eggs after consuming avian plasmas, very small numbers of eggs after consuming human blood cells and relatively large numbers of eggs after feeding on avian blood cells (Fig. 3). Egg hatch rates for *An. gambiae* were also overall lowest after consuming human plasma (Additional file 1: Table S2). *C. quinquefasciatus* laid relatively small numbers of eggs after consuming all plasmas and laid almost no eggs after consuming human blood cells, but laid large numbers of eggs after consuming avian blood cells that were also similar overall to the number of eggs laid after consuming whole avian bloods (Fig. 4). Egg hatch rates for *C. quinquefasciatus* also overall trended lower after feeding on plasmas than blood cells (Additional file 1: Table S2).

### Serum albumins and hemoglobin from different vertebrates variably stimulate egg formation in *Ae. aegypti*

**Fig. 5:**
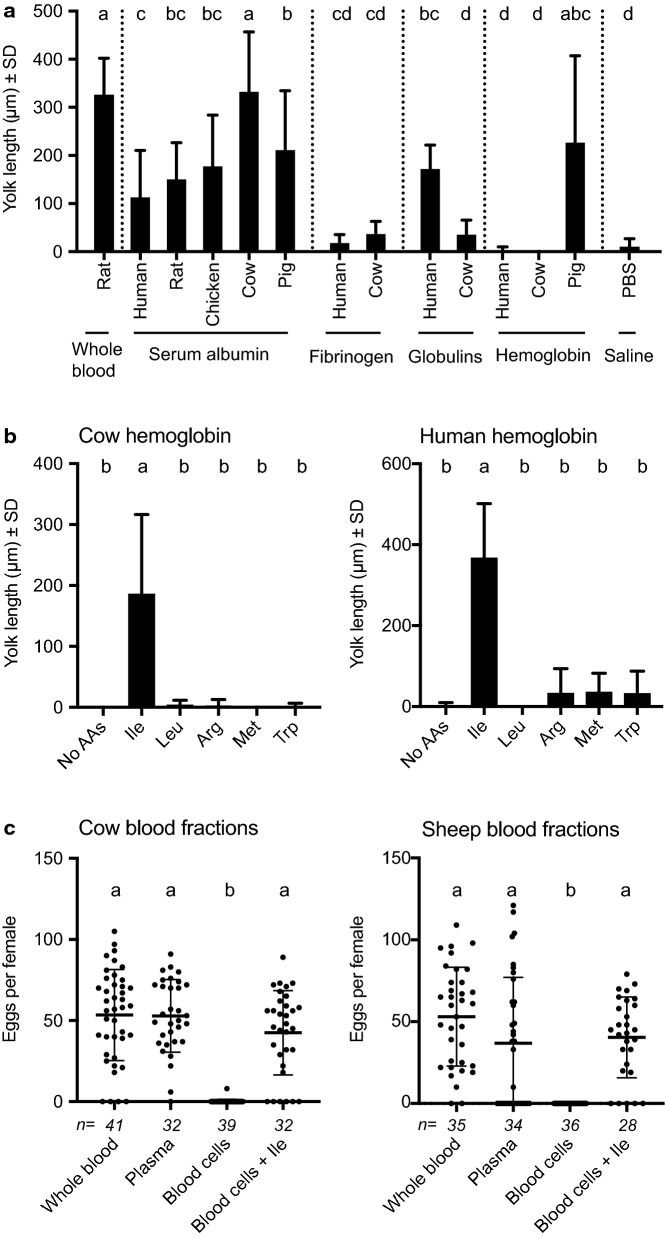
Yolk deposition into oocytes and egg laying by *Ae. aegypti* in response to blood proteins from different vertebrates. **a** Mean yolk length ± SD 48 h after feeding to repletion on blood-derived proteins in PBS-containing ATP from different vertebrates. Each protein was fed to females at 200 mg per ml with whole blood from a rat serving as the positive control and PBS containing ATP serving as the negative control. Small case letters indicate treatments significantly differed from one another after a Kruskal-Wallis and post-hoc Dunn’s test (*p* ≤ 0.05). **b** Mean yolk length ± SD 48 h after feeding to repletion on cow or human hemoglobin with no additional amino acid (AA) or with addition of isoleucine (Ile), leucine (Leu), arginine (Arg), methionine (Met) or tryptophan (Trp). Within each graph different small case letters indicate treatments significantly differed from one another after a Kruskal-Wallis and post-hoc Dunn’s test (*p* ≤ 0.05). **c** Eggs laid per female after feeding to repletion on whole blood, plasma, blood cells or blood cells plus isoleucine from cow or sheep. Numbers above each treatment indicate the total number of replete females. Horizontal bars indicate the mean ± SD. Within each graph different small case letters indicate treatments significantly differed from one another after a Kruskal-Wallis and post-hoc Dunn’s test (*p* ≤ 0.05)

We next assessed how specific blood-associated proteins affected egg formation and key physiological processes that regulate vitellogenesis. We focused these studies on *Ae. aegypti* because: (i) regulation of egg formation in mosquitoes is best understood in this species and (ii) preliminary studies identified issues in feeding purified proteins to *An. gambiae* and *C. quinquefasciatus*, which prevented comparison of outcomes to *Ae. aegypti*. The plasma fraction of vertebrate blood primarily contains three types of proteins (albumin, fibrinogen and globulins) while the cellular fraction by dry weight consists primarily of hemoglobin [85]. We fed *Ae. aegypti* females each of these proteins from at least two vertebrates at 200 mg/ml in PBS plus ATP, which approximates the total protein content of whole human blood. Females that fed to repletion were then dissected 48 h later to determine whether the vitellogenic phase of oogenesis had been activated by measuring yolk deposition, which increases linearly in oocytes when females consume whole blood [18]. Serum albumins from several vertebrates stimulated yolk deposition but only bovine serum albumin (BSA) from cow generated outcomes that were equivalent to females fed whole rat blood (Fig. 5a). Little or no yolk deposition occurred when females consumed fibrinogens or bovine gamma-globulins, while modest yolk deposition occurred after consumption of human gamma-globulins (Fig. 5a).

Vertebrate hemoglobins consist of two alpha and two beta subunits that form heterotetramers with an approximate aggregate mass of 65 kDa [86]. Pig hemoglobin stimulated a variable but often similar yolk deposition response as BSA and whole rat blood, whereas human and cow hemoglobin did not (Fig. 5a). These differences correlated with all essential amino acids being present among the subunits that form the hemoglobins in most mammals including pigs, rats and birds, whereas all subunits forming the non-fetal hemoglobins in primates including humans and even-toed ungulates such as cows and sheep lack isoleucine [87, 88] (Additional file 1: Table S3). Adding isoleucine but not select other essential amino acids to human and cow hemoglobin increased yolk deposition into oocytes to similar levels as pig hemoglobin (Fig. 5b). Feeding females blood cells from cow or sheep resulted in no eggs being laid, but adding isoleucine resulted in females laying similar numbers of eggs as occurred when females were fed whole blood or plasma (Fig. 5c).

### Select other proteins also support egg formation

**Fig. 6:**
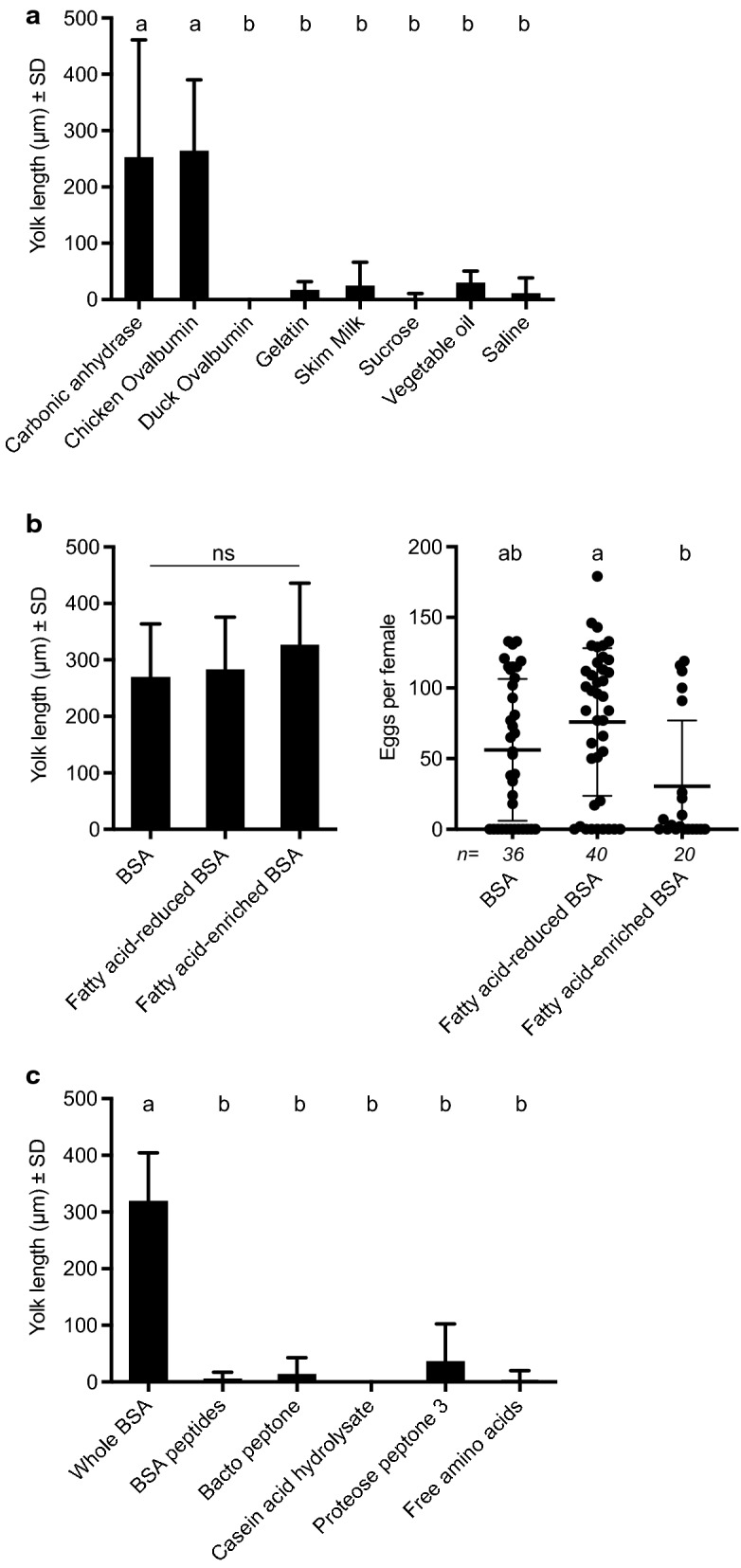
Yolk deposition into oocytes and egg laying by *Ae. aegypti* in response to non-blood proteins and other factors. **a** Mean yolk length ± SD 48 h after feeding to repletion on non-blood proteins, skim milk, sucrose or vegetable oil in PBS containing ATP. Each protein was fed to females at 200 mg/ml while other components were fed at concentrations as indicated in "Methods". PBS containing ATP served as the negative control. Small case letters indicate treatments significantly differed from one another after a Kruskal-Wallis and post-hoc Dunn’s test (*p* ≤ 0.05). **b** Mean yolk length ± SD 48 h or numbers of eggs laid per female after feeding to repletion on BSA, fatty acid depleted BSA or fatty acid-enriched (AlbuMAX II) BSA. For yolk length no significant difference (ns) was detected among treatments after a Kruskal-Wallis test (*p* > 0.05). For eggs per female, numbers above each treatment indicate the total number of replete females, horizontal bars indicate the mean ± SD, and different small case letters indicate treatments significantly differed from one another after a Kruskal-Wallis and post-hoc Dunn’s test (*p* ≤ 0.05). **c** Mean yolk length ± SD 48 h after feeding to repletion on BSA, peptides derived from BSA, other peptide preparations or amino acids that mimic abundance in BSA. Each treatment was fed to females at 200 mg per ml. Small case letters indicate treatments significantly differed from one another after a Kruskal-Wallis and post-hoc Dunn’s test (*p* ≤ 0.05)

We assessed whether any other commonly available proteins that are soluble in PBS at 200 mg/ml also stimulate *Ae. aegypti* to deposit yolk into oocytes. The metalloenzyme carbonic anhydrase is produced in a variety of vertebrate tissues including bovine erythrocytes from which it is purified for commercial sale [89]. This protein and ovalbumin from chicken eggs stimulated yolk deposition, but duck ovalbumin, porcine gelatin and reconstituted skimmed milk powder from cows, which consists primarily of casein [90], did not (Fig. 6a). Sucrose in PBS-containing ATP, lipid (partially hydrogenated vegetable oil) in PBS plus ATP or PBS plus ATP alone were consumed and taken into the midgut by females but little or no yolk was deposited into oocytes (Fig. 6a). Since serum albumins load both fatty acids and steroids [91, 92], we compared females that consumed delipidated or a lipid-enriched BSA (Albumax II) versus standard fraction V BSA. No differences in yolk deposition were detected among these treatments but the number of eggs females laid were highest for females fed delipidated BSA (Fig. 6b). In contrast, females fed standard fraction V BSA digested into peptides using proteinase K or free amino acids at equivalent molarities to BSA at 200 mg/ml stimulated no yolk deposition (Fig. 6c). Several commercially available peptide mixtures (Proteose Peptone 3, Bacto Peptone and Casein Acid Hydrolysate) in PBS plus ATP were also readily consumed by females but stimulated no yolk deposition (Fig. 6c).

### BSA more weakly stimulated trypsin-like activity in the midgut yet comparably stimulated ecdysteroid production by the ovaries compared to blood

**Fig. 7:**
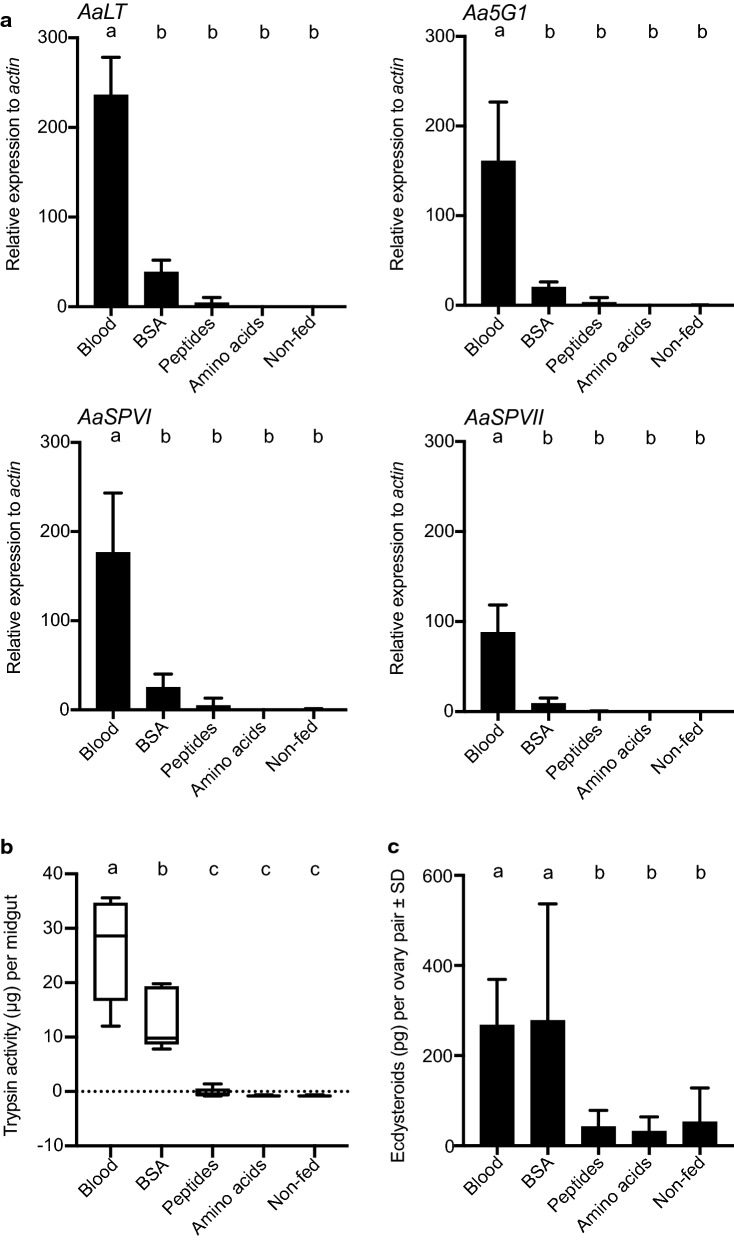
Late-phase serine protease gene expression, midgut trypsin-like activity and ovary ecdysteroid production by *Ae. aegypti* after feeding on blood, BSA or BSA-derived products. **a** rqRT-PCR analysis of *Aa5G1*, *AaLT*, *AaSPVI* and *AaSPVII* expression in midguts 24 h after feeding to repletion on rat blood, BSA, BSA-derived peptides or amino acids that mimic abundance in BSA. Mean relative transcript abundance ± SD for each treatment is normalized to the *actin* gene. Each treatment was replicated four times using midgut samples prepared from different females. Small case letters indicate treatments significantly differed from one another after ANOVA and a post-hoc Tukey-Kramer multiple comparison test (*p*≤0.05). **b** Trypsin-like activity 24 h after the same treatments listed in (**a**). Non-fed females served as the negative control. Horizontal bars indicate the mean ± SD. Small case letters indicate treatments significantly differed from one another after ANOVA and a post-hoc Tukey-Kramer multiple comparison test (*p* ≤ 0.05). **c** Ecdysteroids produced per ovary pair ± SD from females that fed to repletion on the same treatments as in (**a**). Ovaries were collected from females at 24 h post-feeding and ecdysteroid amounts determined by EIA after a 6 h incubation in saline. A minimum of ten independent samples were prepared per treatment with different letters indicating significant differences after ANOVA and a post-hoc Tukey-Kramer multiple comparison test (*p* ≤ 0.05)

As previously noted, bloodmeal consumption by *Ae. aegypti* females stimulates OEH and ILP release from the brain, which regulates bloodmeal digestion by the midgut and ecdysteroid production by the ovaries, which are both essential for egg formation [12–14]. Twelve trypsin-like serine protease (SP) genes are expressed in the midgut with prior studies identifying four (*Aa5G1, AaLT, AaSPVI* and *AaSPVII*) as late-phase SPs that are inducibly expressed by blood feeding and are primarily responsible for bloodmeal digestion [12–14]. *AaSPVI* accounts for most late-phase trypsin-like activity [12–14]. With the potential exception of pig hemoglobin, yolk deposition was more strongly stimulated by BSA than any other blood protein we bioassayed. We therefore asked if BSA induced both high expression of late phase SPs and trypsin-like activity in the midgut, as does whole blood. We also bioassayed females that consumed BSA-derived peptides and the amino acid solution that mimicked the amino acid composition of BSA since neither induced any yolk deposition. BSA induced upregulation of *Aa5G1, AaLT, AaSPVI* and *AaSPVII*, ranging from 20 to 50 times higher relative to peptide-, amino acid- and non-fed females (Fig. 7a). However, expression of these serine proteases was not statistically different among these treatments, in contrast to whole blood, which induced 100–200 times higher expression of these genes relative to non-fed controls (Fig. 7a). BSA also stimulated late trypsin-like activity, but more weakly than whole blood, while no trypsin activity was detected in the midguts of females that consumed peptides or amino acids (Fig. 7b). In contrast, blood and BSA similarly stimulated the ovaries to produce ecdysteroids while peptides and amino acids did not (Fig. 7c).

## Discussion

It has long been recognized that anautogenous mosquitoes blood feed to reproduce [27, 93, 94]. Studies dating back to the 1920s have examined different aspects of how vertebrate blood affects egg formation. However, as noted earlier, most papers in the literature focus on single species of mosquitoes and hosts. Thus, the primary novelty of this study is our comparative approach under standardized assay conditions that examined how: (i) three anautogenous species respond to blood and blood fractions from different vertebrates and (ii) purified blood proteins from different vertebrates affect egg formation and key physiological processes that regulate vitellogenesis in *Ae. aegypti.*

Several laboratory cultures of *Ae. aegypti* including the UGAL strain produce large clutches of eggs after feeding on rat and other rodent bloods, which underlies the common use of these mammals in general rearing [56, 95, 96]. In this study, we identified no differences in egg production between UGAL *Ae. aegypti* that feed on a living rat versus membrane feeders containing whole rat blood unless EDTA was used as an anticoagulant. We thus standardized subsequent assays by using membrane feeders and Na citrate as the anticoagulant. While summaries of the older literature conclude that anautogenous mosquitoes produce more eggs after feeding on vertebrates with nucleated erythrocytes [36, 53], only *C. quinquefasciatus* showed evidence of this in our results. Since *C. quinquefasciatus* preferentially feeds on birds in the field [83, 84, 97], this species may reproductively benefit from avian bloods, which has also been noted for other *Culex* species [30, 32, 33, 35, 98–102]. In contrast, anthropophilic *Ae. aegypti* and *An. gambiae* largely laid similar numbers of eggs across the two mammal and avian blood sources we tested. Our finding that *Ae. aegypti* and *An. gambiae* did not lay more eggs when fed avian blood could reflect an adaptation of our laboratory cultures to mammalian blood, as both species have been reared exclusively on mammalian blood for hundreds of generations. Another possibility is that mosquitoes fed on living hosts are more strongly attracted to odorant and tactile cues from avian hosts and therefore imbibe larger bloodmeals resulting in larger egg clutches. Our artificial membrane feeders are devoid of such cues that might encourage excessive gorging in females.

Contrary to other studies [31, 36, 56, 96, 103], our results show no evidence that *Ae. aegypti* and *An. gambiae* lay smaller clutches after feeding to repletion on whole human blood versus blood from other hosts. However, our results do show dramatic differences in egg formation in response to blood fractions with *Ae. aegypti* and *An. gambiae* laying eggs after consuming mammalian but not avian plasmas. Avian blood cells further stimulated all three mosquito species to lay similar numbers of eggs as whole blood, while human blood cells resulted in females laying few or no eggs. Yet, in the case of *C. quinquefasciatus*, all plasmas stimulated females to lay similar, albeit lower, numbers of eggs than whole blood, while *Ae. aegypti* laid large numbers of eggs after consuming rat blood cells but *An. gambiae* did not. Thus, each of the mosquito species we tested exhibited differences in egg laying across the four vertebrate bloods we tested but the two anthropophiles, *Ae. aegypti* and *An. gambiae*, overall exhibited more similarities to one another than to the ornithophile *C. quinquefasciatus.*

We focused the second part of our study on assessing how the major protein constituents of plasma and blood cells from different vertebrates differentially affect egg formation in *Ae. aegypti*. Pilot experiments revealed that *An. gambiae* and *C. quinquefasciatus* fed poorly when offered meals containing solubilized serum albumins. The response of anopheline species to adenosine nucleotides as phagostimulants is a subject which requires further investigation; we propose that poor feeding on artificial meals not containing blood or blood fractions could be the result of a decreased response to our chosen phagostimulant (ATP) compared to *Ae. aegypti*. In addition to poor feeding, *An. gambiae* in particular exhibited poor survival and viability following serum albumin ingestion. Given these confounding factors, and given that a more expansive molecular toolkit exists for the model organism *Ae. aegypti,* we chose not to use *An. gambiae* or *C. quinquefasciatus* in our studies of how purified blood proteins affect egg formation and the physiological processes that regulate vitellogenesis.

Hemoglobin accounts for ~90% of the total protein in vertebrate blood and thus is far more abundant than any plasma protein [54]. We therefore standardized the concentration of each protein we tested (200 mg/ml) in feeding assays to reflect total protein in human blood rather than the actual abundance of each component. As previously noted, several studies report that BSA and whole blood comparably stimulate yolk deposition into primary follicles [67–74]. In contrast, our results show that serum albumins from other vertebrates more weakly stimulate yolk deposition than BSA, which was unanticipated given: (i) lyophilized plasmas of several mammals were recently reported to stimulate high-level egg production in *Ae. aegypti* [66] and (ii) mammalian serum albumins share high amino acid identity (74–76%) and have similar crystal structures [104, 105]. Our results further indicate that fibrinogens and globulins from two mammals (human, cow) stimulate low rates of yolk deposition. In contrast, hemoglobin from pig stimulates similar yolk deposition rates to whole rat blood and BSA while human and cow hemoglobin stimulates no yolk deposition. Thus, our results indicate that purified blood proteins differentially stimulate yolk deposition and that UGAL *Ae. aegypti* also differentially respond to homologs of the same protein from different vertebrates. That chicken ovalbumin stimulates yolk deposition indicates certain proteins absent in vertebrate blood can stimulate egg formation. Other studies also report variable oogenic responses by different mosquito species fed egg albumin, gelatin, skim milk, agar, soy infant formula and hemolymph from other insects [55, 62, 106–110].

Since albumins bind a range of lipids and other ligands [91, 111], we reasoned this variable potentially contributes to the differences in yolk deposition we see among vertebrate albumins. A previous study also reported that addition of LDL and cholesterol to a BSA-based artificial diet increased fecundity in *Ae. aegypti* [70]. However, we detected no differences in yolk deposition between BSAs enriched or depleted for bound lipids and lipid-enriched BSA resulted in females laying fewer eggs. It is nevertheless possible that factors binding to serum albumins potentially contribute to the between-species differences in albumins we observed. On the other hand, our finding that females produce no mature eggs after consuming BSA-derived peptides, peptides from other proteins or free amino acids mimicking the composition of BSA suggest proteins must be of a minimum size for digestion and absorption and that small peptides and amino acids are potentially excreted too rapidly to be efficiently absorbed. This suggestion would also be consistent with studies showing that slow, continuous perfusion of essential amino acids into the hemocoel of mosquitoes promotes egg formation while single injections of amino acids do not [52, 108, 112]. We also note that our findings are not wholly in accord with two early reports that peptides and amino acids stimulated very low-level, but nonzero, egg formation in *Ae. aegypti* [113, 114].

Previous studies noted that isoleucine deficiencies in the blood of certain vertebrates correlate with reductions in egg production by anautogenous mosquitoes [51, 54, 103]. In contrast, our use of purified proteins definitively indicates that *Ae. aegypti* produces no mature eggs when fed hemoglobins lacking isoleucine but large numbers of eggs when fed hemoglobins that contain the essential amino acid isoleucine or when fed an isoleucine-deficient hemoglobin that is isoleucine-supplemented. The effect of isoleucine deficiency was previously attributed to essential amino acid balance being important for egg production [51, 55, 96]. However, amino acids function as nutritional signals in regulating a number of cellular processes through the TOR and insulin signaling pathways [115–117]. Amino acids also regulate translation initiation with the absence of any essential amino acid resulting in suppression of protein synthesis [118, 119]. Thus, more complex mechanisms than amino acid balance likely underlie the severe defects in egg formation that occur when female mosquitoes feed on isoleucine-deficient hemoglobins.

Since BSA strongly stimulates egg formation in *Ae. aegypti*, we asked if this protein and whole blood similarly stimulate two physiological processes required for vitellogenesis: late-stage serine protease gene expression in the midgut and ecdysteroid production by the ovaries. BSA did induce the expression of four proteases with known roles in bloodmeal digestion, *Aa5G1, AaLT, AaSPVI* and *AaSPVII*; however, levels of expression and trypsin-like activity were roughly half the levels measured in blood-fed females. Hundreds of serine proteases are expressed in *Ae. aegypti* [14], and midgut-localized proteases include not only trypsin-like enzymes but also chymotrypsins and two major classes of exopeptidases, aminopeptidases and carboxypeptidases [13, 120, 121]. Therefore, it is possible that BSA is primarily digested by enzymes other than the trypsin-like proteases we surveyed. In contrast, BSA and whole blood comparably stimulate ecdysteroid production by the ovaries, which is consistent with BSA and blood-fed females laying similar numbers of eggs [65, 68, 71].

## Conclusions

Three species of anautogenous mosquitoes variably produced eggs in response to whole vertebrate blood, blood fractions or specific proteins from different vertebrates. For the most abundant protein in blood, hemoglobin, major differences in egg formation by *Ae. aegypti* were clearly due to isoleucine deficiency of this protein from select vertebrates, whereas the underlying basis for serum albumins differentially affecting egg formation remained unclear. Physiological assays in *Ae. aegypti* further showed that blood and BSA differentially stimulate digestive enzyme activity but comparably stimulate ecdysteroidogenesis.

## Supplementary Information

**Additional file 1: Table S1.** Amino acid amounts added to PBS to equal their abundance in BSA at 200 mg/ml. **Table S2.** Proportion of hatching eggs for mosquitoes fed whole blood or fractions from four vertebrate hosts. **Table S3. **Amino acid compositions of major hemoglobin subunits of adult-stage vertebrates.

## Data Availability

All essential data for this study are presented in the main text and supplementary information section. Data sets are freely available from the corresponding author upon request.

## Abbreviations

20E: 20-Hydroxyecdysone
AA: Amino acid
ATP: Adenosine triphosphate
BSA: Bovine serum albumin
ECD: Ecdysone
EDTA: Ethylenediaminetetraacetic acid
EIA: Enzyme-linked immunosorbent assay
ILP: Insulin-like peptide
IR: Insulin receptor
OEH: Ovary ecdysteroidogenic hormone
OEHR: OEH receptor
PBS: Phosphate-buffered saline
SD: Standard deviation
SP: Serine protease
TOR: Target of rapamycin
Vg: Vitellogenin
