# Supplementary material for: Whole blood and blood components from vertebrates differentially affect egg formation in three species of anautogenous mosquitoes
Source: Parasit Vectors. 2021 Feb 24;14:119. doi: 10.1186/s13071-021-04594-9 (PMC7905675; doi:10.1186/s13071-021-04594-9)
Supplement: Supplementary file 1 — Additional file 1: Table S1. Amino acid amounts added to PBS to equal their abundance in BSA at 200 mg/ml. Table S2. Proportion of hatching eggs for mosquitoes fed whole blood or fractions from four vertebrate hosts. Table S3. Amino acid compositions of major hemoglobin subunits of adult-stage vertebrates. [file 13071_2021_4594_MOESM1_ESM.docx]

**Table S1. Amino acid amounts added to PBS to equal their abundance in BSA at 200 mg/ml.**

|  | Amino acid | AA formula composition (g/L) |
| --- | --- | --- |
| Essential amino acids | Phe | 14.3 |
|  | His | 7.6 |
|  | Ile | 5.7 |
|  | Lys | 25.3 |
|  | Leu | 24.6 |
|  | Met | 2.1 |
|  | Arg | 13.0 |
|  | Thr | 11.7 |
|  | Val | 12.9 |
|  | Trp | 1.8 |
| Non-essential amino acids | Ala | 12.1 |
|  | Cys | 12.2 |
|  | Asp | 15.4 |
|  | Glu | 25.0 |
|  | Gly | 3.6 |
|  | Asn | 5.3 |
|  | Pro | 9.3 |
|  | Gln | 8.4 |
|  | Ser | 9.7 |
|  | Tyr* | 5.0* |

L-enantiomer forms of each amino acid were used, and the solution

was adjusted to neutral pH using NaOH.

*The amount of tyrosine in the formula was halved from its

relative abundance in BSA to allow full solubilization.

**Table S2. Proportion of hatching eggs for mosquitoes fed whole blood or fractions from four vertebrate hosts.**

| Proportion hatched (total eggs laid)^1^ | | | | | |
| --- | --- | --- | --- | --- | --- |
| *Aedes aegypti* | | | | |  |
|  | Human | Rat | Chicken | Turkey |  |
| Whole blood^1^ | 0.49 (2201) | 0.33 (2231) | 0.16 (1538) | 0.70 (2611) | *p* < 0.0001 |
| Plasma^2^ | 0.39 (421) | 0.47 (936) | NA | NA | *p* < 0.0001 |
| Blood cells^3^ | 0.21 (129) | 0.75 (1906) | 0.63 (2189) | 0.68 (1025) | *p* < 0.0001 |
|  |  |  |  |  |  |
| *Anopheles gambiae* | | | | |  |
|  | Human | Rat | Chicken | Turkey |  |
| Whole blood^4^ | 0.41 (870) | 0.43 (987) | 0.30 (165) | 0.52 (626) | *p* < 0.0001 |
| Plasma^5^ | 0.47 (719) | 0.62 (631) | NA | NA | *p* < 0.0001 |
| Blood cells^6^ | 0.14 (53) | 0.33 (94) | 0.33 (2290) | 0.34 (2475) | *p* = 0.0022 |
|  |  |  |  |  |  |
| *Culex quinquefasciatus* | | | | |  |
|  | Human | Rat | Chicken | Turkey |  |
| Whole blood^7^ | 0.44 (2000) | 0.59 (3297) | 0.33 (2624) | 0.48 (5125) | *p* < 0.0001 |
| Plasma^8^ | 0.26 (1706) | 0.33 (477) | 0.33 (460) | 0.15 (337) | *p* < 0.0001 |
| Blood cells^9^ | 0.32 (25) | 0.65 (378) | 0.47 (4043) | 0.35 (3530) | *p* < 0.0001 |
|  |  |  |  |  |  |

^1^NA indicates no eggs were laid by females. Statistical significance among treatment means is indicated to the right of each row (four-group $X^{2}$ test).

**Table S3. Amino acid compositions of major hemoglobin subunits of adult-stage vertebrates.**

|  |  | Alpha subunits | | | | | | | | |  | Beta subunits | | | | | | | |  | Delta subunit |
| --- | --- | --- | --- | --- | --- | --- | --- | --- | --- | --- | --- | --- | --- | --- | --- | --- | --- | --- | --- | --- | --- |
|  |  | Human | Rat | Cow | Sheep | Pig | Chicken | | Turkey | |  | Human | Rat | | Cow | Sheep | Pig | Chicken | Turkey |  | Human |
|  |  | *HBA-1/2* | *HBA-1/2* | *HBA-1/2* | *HBA-1/2* | *HBA* | *HBAA* | *HBAD* | *HBAA* | *HBAD* |  | *HBB* | *HBB-1* | *HBB-2* | *HBB* | *HBB* | *HBB* | *HBB* | *HBB* |  | *HBD* |
| A | Ala | 21 | 17 | 20 | 17 | 20 | 18 | 17 | 17 | 17 |  | 15 | 16 | 17 | 16 | 16 | 14 | 16 | 16 |  | 15 |
| ­C | Cys | 1 | 3 | 0 | 1 | 1 | 2 | 1 | 2 | 1 |  | 2 | 2 | 2 | 1 | 1 | 1 | 3 | 3 |  | 2 |
| D | Asp | 8 | 10 | 8 | 9 | 10 | 7 | 7 | 7 | 7 |  | 7 | 9 | 7 | 9 | 8 | 8 | 6 | 7 |  | 7 |
| E | Glu | 4 | 4 | 5 | 1 | 10 | 6 | 8 | 6 | 8 |  | 8 | 5 | 5 | 8 | 9 | 8 | 7 | 6 |  | 7 |
| F | Phe | 7 | 7 | 7 | 7 | 7 | 7 | 7 | 7 | 7 |  | 8 | 7 | 7 | 10 | 9 | 8 | 8 | 8 |  | 8 |
| G | Gly | 7 | 10 | 9 | 11 | 10 | 9 | 7 | 9 | 7 |  | 13 | 14 | 13 | 11 | 11 | 13 | 8 | 8 |  | 13 |
| H | His | 10 | 10 | 10 | 9 | 11 | 10 | 6 | 10 | 6 |  | 9 | 10 | 10 | 6 | 9 | 8 | 7 | 7 |  | 7 |
| I | Ile | 0 | 3 | 0 | 0 | 0 | 7 | 2 | 8 | 2 |  | 0 | 4 | 4 | 0 | 0 | 1 | 7 | 7 |  | 0 |
| K | Lys | 11 | 12 | 11 | 10 | 11 | 12 | 11 | 12 | 11 |  | 11 | 12 | 12 | 13 | 10 | 11 | 10 | 10 |  | 11 |
| L | Leu | 18 | 15 | 20 | 19 | 19 | 15 | 15 | 15 | 15 |  | 18 | 17 | 17 | 17 | 17 | 19 | 18 | 18 |  | 18 |
| M | Met | 3 | 3 | 2 | 2 | 1 | 2 | 4 | 2 | 4 |  | 2 | 3 | 3 | 3 | 2 | 2 | 2 | 2 |  | 3 |
| N | Asn | 4 | 3 | 3 | 5 | 5 | 4 | 6 | 4 | 6 |  | 6 | 8 | 8 | 7 | 6 | 9 | 7 | 7 |  | 8 |
| P | Pro | 7 | 6 | 6 | 6 | 6 | 6 | 5 | 6 | 5 |  | 7 | 5 | 4 | 4 | 3 | 4 | 5 | 5 |  | 6 |
| Q | Gln | 1 | 2 | 1 | 1 | 2 | 2 | 8 | 2 | 58 |  | 3 | 3 | 4 | 3 | 5 | 5 | 5 | 5 |  | 5 |
| R | Arg | 3 | 3 | 3 | 4 | 3 | 3 | 4 | 3 | 4 |  | 3 | 3 | 3 | 4 | 5 | 5 | 6 | 6 |  | 4 |
| S | Ser | 11 | 11 | 13 | 12 | 10 | 6 | 8 | 6 | 8 |  | 5 | 6 | 7 | 5 | 6 | 6 | 7 | 8 |  | 6 |
| T | Thr | 9 | 9 | 8 | 10 | 7 | 10 | 7 | 9 | 7 |  | 7 | 4 | 5 | 6 | 5 | 2 | 7 | 6 |  | 5 |
| V | Val | 13 | 10 | 12 | 13 | 12 | 12 | 12 | 13 | 12 |  | 18 | 14 | 14 | 18 | 19 | 19 | 12 | 12 |  | 17 |
| W | Trp | 1 | 1 | 1 | 1 | 1 | 0 | 1 | 0 | 1 |  | 2 | 2 | 2 | 2 | 2 | 2 | 4 | 4 |  | 2 |
| Y | Tyr | 3 | 3 | 3 | 3 | 2 | 4 | 5 | 4 | 5 |  | 3 | 3 | 3 | 2 | 2 | 2 | 2 | 2 |  | 3 |
|  |  |  |  |  |  |  |  |  |  |  |  |  |  |  |  |  |  |  |  |  |  |

Total amino acid residues in hemoglobin subunits, including any non-allelic chain variants, are indicated with essential amino acids shaded gray. Hemoglobin subunits prevalent in embryonic or fetal stages, *i.e*., gamma, epsilon, mu, pi, theta, and zeta, are not reported due to their absence in adult-stage vertebrates. In contrast, the fetal hemoglobin subunit delta (HBD) is shown for human hosts: hemoglobin A (HbA), comprised of two alpha and two beta subunits, accounts for 97% of hemoglobin in adults, but hemoglobin A2 (HbA2), comprised of two alpha and two delta chains, accounts for the remaining 3%.
